# Distinct long-term disease activity trajectories differentiate early on treatment with etanercept in both rheumatoid arthritis and spondylarthritis patients: a prospective cohort study

**DOI:** 10.1007/s00296-023-05455-7

**Published:** 2023-10-10

**Authors:** Irini Flouri, Panagiota Goutakoli, Argyro Repa, Antonios Bertsias, Nestor Avgoustidis, Anastasios Eskitzis, Sofia Pitsigavdaki, Eleni Kalogiannaki, Maria Terizaki, George Bertsias, Prodromos Sidiropoulos

**Affiliations:** 1 Rheumatology, Clinical Immunology and Allergy Department, Medical School, University of Crete, Heraklion, Greece; 2 Laboratory of Rheumatology, Autoimmunity and Inflammation, Medical School, University of Crete, Heraklion, Greece and Institute of Molecular Biology and Biotechnology, Foundation for Research and Technology Hellas (FORTH), Heraklion, Greece

**Keywords:** Tumor necrosis factor inhibitor, Rheumatoid arthritis, Axial spondyloarthritis, Psoriatic arthritis, Etanercept, Disease activity trajectories, Drug survival

## Abstract

To characterize disease activity trajectories and compare long-term drug retention between rheumatoid (RA) and spondylarthritis (SpA) patients initiating tumor necrosis factor inhibitor (TNFi) treatment (etanercept). Prospective observational study of RA, axial (AxSpA) and peripheral SpA (PerSpA) patients initiating etanercept during 2004–2020. Kaplan–Meier plots were used for drug retention comparisons and multivariable Cox regression models for predictors of discontinuation. Long-term disease activity trajectories were identified by latent class growth models using DAS28-ESR or ASDAS-CRP as outcome for RA and AxSpA respectively. We assessed 711 patients (450 RA, 178 AxSpA and 83 PerSpA) with a median (IQR) follow-up of 12 (5–32) months. At 5 years, 22%, 30% and 21% of RA, AxSpA and PerSpA patients, respectively, remained on therapy. Etanercept discontinuation was independent of the diagnosis and was predicted by gender and obesity in both RA and SpA groups. Four disease activity (DA) trajectories were identified from 6th month of treatment in both RA and AxSpA. RA patients in remission-low DA groups (33.7%) were younger, had shorter disease duration, fewer comorbidities and lower baseline disease activity compared to moderate (40.6%) & high DA (25.7%) groups. In AxSpA 74% were in inactive-low DA and they were more often males, non-obese and had lower number of comorbidities compared to higher ASDAS-CRP trajectories. In RA and AxSpA patients, disease activity trajectories revealed heterogeneity of TNFi treatment responses and prognosis. Male gender, lower baseline disease activity and fewer comorbidities, characterize a favourable outcome in terms of disease burden accrual and TNFi survival.

**Supplementary Information:**

The online version contains supplementary material available at 10.1007/s00296-023-05455-7.

## Introduction

Rheumatoid arthritis (RA) and spondylarthritis (SpA) (axial or peripheral) are chronic systemic inflammatory arthritides (IA), characterized by pathogenetic and phenotypic heterogeneity and diverse responses to treatments [1, 2]. Although distinct molecular and cellular pathways are involved in the pathogenesis of RA and SpA, inhibition of TNFα has been successfully proved as a common therapeutic target. However, response to treatment with TNFα inhibitors (TNFis) is mostly unpredictable, while patients’ disease course varies.

Recently, methods to characterize groups of patients with different disease activity trajectories while on treatment with biologics have gained popularity. Trajectory modelling identifies patient clusters according to the similarity of their disease progression in time, a preferred method compared to the dichotomous (either a responder or a non-responder) indices measuring response as a snap-shot at a chosen timepoint. However, studies of disease activity trajectories using data from randomised clinical trials (RCTs) [3–6] or form observational settings [7–12] are still sparse and mostly include early RA patients and short-term follow-up. Relevant literature for SpA is more limited and performed in groups of patients with early AxSpA [13, 14] or heterogenous populations (early and established AS) receiving various treatments (NSAIDS or biologics) [15]. Furthermore, studies comparing long-term disease activity trajectories between RA and SpA are not available.

Most of the aforementioned studies analyze patients receiving TNFis as a group. However, differences in clinical efficacy and drug survival of different TNFis in RA or SpA have been shown in previous works [16, 17]. Thus, analyzing patients on a single TNFi may exclude any differential effect of other biologics on long-term treatment outcomes. Etanercept is one of the first TNFα inhibitors approved for the treatment of IA and has been shown to be effective in RA and SpA patients both in early and established disease [18, 19].

In the present analysis, we aimed to compare in the same research setting long-term drug survival and disease activity trajectories of RA and SpA patients treated with the etanercept, thus excluding any differential effect of different biologics on treatment outcomes. Furthermore, we sought to determine predictors of good response and longer etanercept survival in the two diseases and we investigated the impact of belonging to different disease activity trajectories in terms of long-term patient function and the cumulative incidence of serious adverse events.

## Methods

### Cohort

The present study was based on the University of Crete Rheumatology Clinic Registry (UCRCR), a single center prospective cohort study. Patients ≥ 18 years old with IA are included in UCRCR at the time of initiation of the first biologic or targeted synthetic (b-/ts)-DMARD and they are prospectively followed irrespectively of treatment switches for as long as they receive b-/ts-DMARD therapy. Biological treatment initiation, as well as all treatment decisions (bDMARD selection, co-medication, dosage adjustments/switches) are made by the attending rheumatologists based on clinical assessments, national guidelines and patient’s preferences. According to the national guidelines, patients are considered candidates for biologic treatment if they have active disease (in RA defined as DAS28 > 3.2 and in AxSpA ASDAS > 2.1) and have failed previous treatment with at least one disease modifying drug (in RA and perSpA) or two NSAID courses (in AxSpA). According to the protocol, data on demographics, disease characteristics, comorbidities, disease activity, function and quality of life are collected at bDMARD initiation and every 3–6 months for the first 2 years and yearly thereafter [20]. Treatment discontinuations are registered prospectively and classified by the treating physician as due to treatment inefficacy (primary and secondary), adverse event(s), patient decision, pregnancy, disease remission, or other reasons. In cases of loss of follow-up of a patient for more than one and a half years, the patient is reported as “lost to follow-up” at the date of the last recorded follow-up visit.

In the present study, we included patients who received at least one etanercept subcutaneous injection between 1-2004 and 31–12-2020 for the diagnosis of either RA, axial (AxSpA) or peripheral SpA (PerSpA). Diagnosis was based on the judgment of the treating rheumatologist, but the fulfilment of the classification criteria for the respective diagnosis (ACR/EULAR 2010 RA Classification Criteria, ASAS Axial and Peripheral SpA criteria, or PsA CASPAR criteria) was also recorded. Ninety nine percent of the included RA patients, 93% of AxSpA and 100% of peripheral SpA patients fulfilled the aforementioned classification criteria. No specific exclusion criteria were applied. Patients were followed until discontinuation of etanercept, death, loss of follow-up, or 31 May 2021. All patients provided a written informed consent at inclusionin the registry according to the Declaration of Helsinki.

### Outcome variables

Disease activity in RA and SpA patients having peripheral disease was measured by Disease Activity Score in 28 joints based on erythrocyte sedimentation rate (DAS28-ESR) [21], Simplified Disease Activity Index (SDAI) [22] and Clinical Disease Activity Index (CDAI) [23], calculated by the following formulas:

$${\text{DAS}}28 - {\text{ESR }} = \, 0.56 * \surd \left( {28{\text{tender joint count}}} \right) \, + \, 0.28 * \surd \left( {\text{Swollen joint count}} \right) \, + \, 0.70 * \ln \left( {{\text{ESR}}} \right) \, + \, 0.014 * {\text{Patient global assessment}}$$

$${\text{SDAI }} = {\text{Swollen joint count }} + {\text{ Tender joint count }} + {\text{ Physician}}'{\text{s}}\;{\text{global assessment}} + {\text{ Patient's}}\;{\text{global assessment}} + {\text{ C}} - {\text{reactive protein in }}{{{\text{mg}}} \mathord{\left/ {\vphantom {{{\text{mg}}} {{\text{dl}}}}} \right. \kern-\nulldelimiterspace} {{\text{dl}}}}$$

$${\text{CDAI}} = {\text{Swollen joint count}} + {\text{Tender joint count}} + {\text{Physician}}^{\prime}{\text{s}}\;{\text{global assessment}} + {\text{Patient}}^{\prime}{\text{s}}\;{\text{global assessment}}$$

Functional status of these patients was assessed by the modified Health Assessment Questionnaire (mHAQ) [24], a short version of the original 20-question HAQ. Disease activity and function in AxSpA patients was measured using the Ankylosing Spondylitis Disease Activity Score based on C-reactive protein (ASDAS-CRP), Bath Ankylosing Spondylitis Disease Activity Score (BASDAI) and Bath Ankylosing Spondylitis Index (BASFI) [25]. BASDAI is a self-administered patient questionnaire assessing fatigue, axial and peripheral symptoms, enthesopathy and duration and intensity of morning stiffness using visual analogue scales (VAS in centimeters, 0–10) [25]. ASDAS has been developed to improve the objectivity of this index and includes the questions of BASDAI concerning the level of axial and peripheral symptoms and the duration of morning stiffness, but also the level of acute phase reactants – either ESR or CRP—and an overall global assessment in VAS (0–10) [26].

Disease activity status in RA and Axial SpA was defined based on published cut-offs of DAS28 [High disease activity (HDA) > 5.1, moderate disease activity (MDA) 3.2–5.1, low disease activity (LDA) ≥ 2.6 and < 3.2 and remission (REM) < 2.6] and ASDAS (very HDA > 3.5, HDA 2.1—3.5, LDA < 2.1 and ≥ 1.3 and REM < 1.3) respectively [27, 28]. Moreover, information on all adverse events [“Medical Dictionary for Regulatory Activities” (MedDRA)-coded and categorized according to severity and any relation to therapy] were analyzed.

Drug retention was calculated as the time period between the first prescription of etanercept and the date of the first missed dose of the drug, death, or 31/05/2021. Temporary treatment interruptions of < 6 months (e.g. due to adverse events, surgeries, loss of insurance, etc.) were allowed. Patients lost to follow-up were censored at their last recorded visit.

## Statistical analysis

Data are presented with standard descriptive statistics and differences between groups were analyzed using One-way-Anova (for normally distributed data), the non-parametric Kruskal–Wallis test (for non-normally distributed data) and the Pearson’s chi-square test as appropriate. Post-hoc comparisons were performed using the Tuckey-Kramer test (for normally distributed data), Dunn’s test with Benjamini–Hochberg stepwise adjustment (for non-normally distributed data) [29] and Pairwise Z-test with Bonferroni correction. Kaplan–Meier plots with log-rank tests were used to explore differences in drug survival between the three different diagnoses. For the analysis of time to treatment discontinuation due to ineffectiveness, discontinuations due to other reasons were treated as censored observations. Multivariate Cox and logistic regression models were employed to assess for factors associated with etanercept discontinuation and patient response to therapy, including baseline demographics, disease and patient characteristics. Baseline variables with < 30% missing values were first tested in univariable Cox regression analyses and non-collinear factors with a p-value < 0.15 were included in the multivariable model. Variables with least significance were then excluded stepwise until only variables with a p value < 0.15 remained in the model.

Distinct trajectories of disease activities were identified using latent class growth models (LCGM). For this analysis we used the DAS28-ESR scores (for RA patients) and ASDAS-CRP (for AxSpA patients) and time polynomials (linear, quadratic, cubic) as covariates. For RA patients the time period used was from 0 to 48 months and for AxSpA patients was from 0 to 24 months, due to low number of disease activity scores measured thereafter. PerSpA patients were excluded in this analysis due to the low number of patients. The adjusted Bayesian information Criterion (BIC) was used in order to identify the best fitting model with smaller values indicating a better model fit. Four-group cubic models were identified at the ones with the best model fit. Additional analysis of screen plots for the curves generated from the within sum of squares (WSS) and the η^2^ coefficient, which is quite similar to the R^2^, or the proportional reduction of error (PRE) coefficient was performed [30]. Such analysis is typically used in k-means cluster analyses when the number of clusters is unknown. Our analysis indicated that adding more than 4 groups offers little in the reduction of WSS values or the respective increase of the η^2^ value (Supplementary Figure). We also performed univariate analysis in order to identify potential differences in baseline characteristics between the trajectory groups. For RA patients we additionally performed a linear mixed model plot of HAQ values over time using the trajectory groups produced above as a covariate. Trajectory analyses and plots were performed using the “Traj” plugin for STATA [29, 31]. Intention-to-treat analysis was carried throughout. All analyses were performed using STATA version 16 and Statistical Package for Social Sciences version 22 (SPSS, SPSS Inc). P-values of 0.05 (two- tailed) were considered statistically significant.

## Results

### Baseline patients’ characteristics

We analyzed 711 patients (RA = 450, AxSpA = 178 and PerSpA = 83) starting etanercept and prospectively followed for 1371 patient-years. The median (interquartile range) follow-up [12 (5.2–32) months] was comparable between the different diagnoses. As expected, there was a heterogeneity in baseline demographics, disease characteristics and co-administered treatments between the three diagnosis groups (Table 1). RA patients were older, had more comorbidities and received etanercept less often as monotherapy, while disease activity indices (DAS28-ESR and ASDAS-CRP) as well as patients’ perception of disease activity status (VAS global) were high and comparable between RA and SpA. Of note, PerSpA patients had lower disease activity status and better function as compared to the two other groups.

**Table 1:** Baseline patient demographics, disease characteristics and treatments

	Valid	Total (n = 711)	RA (n = 450)	AxSpA (n = 178)	PerSpA (n = 83)	p-value
Women, N (%)	711	482 (68)	370 (82)	62 (35)	50 (60)	** < 0.001**
Age	711	56 (46–66)	61.5 (53–70)	46 (37–55)	49 (34–60)	** < 0.001**
Disease duration from diagnosis	485	2.1 (0.6–6.2)	2.6 (0.9–6.5)	1.1 (0.1–5.7)	1.2 (0.2–4.4)	** < 0.001**
Symptom duration, years	656	7.4 (3.3–15.1)	6.3 (3.0–12.6)	12.8 (6.3–22)	5.8 (1.8–11.9)	** < 0.001**
Treatment line, N (%): 1st	711	394 (55)	264 (59)	83 (47)	47 (57)	**0.003**
2nd	711	213 (30)	119 (26)	74 (42)	20 (24)	
≥ 3rd	711	104 (15)	67 (15)	21 (12)	16 (19)	
Nr of previous csDMARDs	711	2 (1–3)	2 (1–3)	1 (0–2)	1 (1–2)	** < 0.001**
Co-administered MTX, N(%)	711	399 (56)	284 (63)	65 (37)	50 (60)	** < 0.001**
Monotherapy, N (%)	711	185 (26)	60 (13)	101 (57)	24 (29)	** < 0.001**
Ongoing corticosteroids, N (%)	711	195 (27)	153 (34)	22 (12)	20 (24)	** < 0.001**
Total Comorbidities Count	543	2 (1–4)	3 (1–4)	1 (0–2)	2 (1–4)	** < 0.001**
RDCI	543	1 (0–2)	1 (1–2)	0 (0–1)	1 (0–2)	** < 0.001**
Ever smokers, N (%)	490	236 (33)	124 (39)	84 (72)	28 (52)	** < 0.001**
Obesity (BMI > 30), N (%)	568	210 (37)	156 (41)	30 (22)	24 (44)	** < 0.001**
Years of education: 0 to 6	335	199 (53)	159 (60)	27 (35)	13 (41)	** < 0.001**
7 to 12	335	116 (31)	74 (28)	29 (37)	13 (41)	
> 12	335	61 (16)	33 (12)	22 (28)	6 (19)	
Residence, N (%): Rural	602	285 (47)	193 (50)	69 (48)	23 (32)	**0.003**
Employed, N (%)	492	146 (30)	62 (19)	60 (53)	24 (48)	** < 0.001**
DAS28 – ESR	428	5.7 (4.8–6.5)	5.8 (5.0–6.5)	–	4.6 (3.6–5.5)	** < 0.001**
SDAI	257	34 (26–43)	35 (28–44)	–	21 (16–36)	**0.006**
CDAI	350	34 (24–42)	36 (27–44)	–	19 (14–29)	** < 0.001**
ASDAS-CRP	103	3.6 (3.0–4.3)	–	3.6 (3.0–4.3)	–	
BASDAI (0–10)	110	5.9 (4.8–7.1)	–	5.9 (4.8–7.1)	–	
Swollen JC (0–28)	572	8 (2–12)	10 (6–13)	0 (0–3)	3 (0–8)	** < 0.001**
Tender JC (0–28)	571	7 (2–13)	9 (5–14)	1 (0–3)	3 (0–8)	** < 0.001**
VAS global (0–100)	560	70 (60–80)	70 (60–80)	70 (60–80)	70 (60–80)	0.276
VAS pain (0–100)	456	70 (60–80)	76 (60–80)	70 (50–80)	70 (56–80)	0.144
VAS physician (0–100)	498	75 (65–80)	78 (70–80)	75 (63–80)	75 (63–75)	**0.008**
CRP (mg/dl)	425	0.5 (0.3–1.4)	0.4 (0.3–1.1)	1.1 (0.4–2.4)	0.6 (0.3–1.7)	** < 0.001**
ESR (mm/h)	564	25 (15–42)	26 (15–40)	25 (15–47)	25 (15–42)	0.895
HAQ (0–3)	333	0.8 (0.5–1.3)	0.9 (0.5–1.3)	0.8 (0.6–1.2)	0.5 (0.2–1.0)	**0.039**
BASFI (0–10)	96	6.4 (4.3–7.8)	–	6.4 (4.3–7.8)	–	

All values are medians (IQR) unless otherwise specified. Differences between groups were analyzed using the Kruskal–Wallis and chi-square tests as appropriate. *RA* rheumatoid arthritis; *AxSpA* axial spondyloarthritis; *PerSpA* peripheral SpA; *BMI* body mass index; *RDCI* rheumatic disease comorbidity index; *csDMARDs* conventional synthetic disease modifying anti-rheumatic drugs; *bDMARDs* biologic DMARDs; *ΜΤΧ* Methotrexate; *DAS28* disease activity score using 28 joints; sdai simplified disease activity index; CDAI clinical disease activity index; *ASDAS* ankylosing spondylitis disease activity score; *CRP* C-reactive protein, *ESR* erythrocyte sedimentation rate; *BASDAI* bath ankylosing spondylitis disease activity index; *JC* joint count, *VAS* visual analogue scale; *HAQ* health assessment questionnaire; *BASFI* bath ankylosing spondylitis functional index

### Long-tern etanercept survival and baseline predictors

Overall, 466 (65.5%) patients discontinued etanercept. Approximately half of the stops (55.8%) occurred within the first year of therapy. Treatment inefficacy was the most frequent cause of discontinuation (70.2% of cases), while stops due to adverse events were infrequent (19%) across all diagnoses (Supplementary Table 1 and Supplementary Table 2 for a description of all adverse events during follow-up). The overall estimated 1-year retention rates of etanercept in RA, AxSpA and PerSpA were 57.5%, 67% and 56.8% respectively, while the respective 5-year retention rates were 22%, 30% and 21% (log-rank p = 0.204 and p = 0.006 for the 1-year and 5-year comparisons respectively). When considering only discontinuations due to inefficacy, 1-year (5-year) estimated retention rates were 64.8% (32%), 77.5% (48%) and 74.5% (42%) respectively (log-rank p < 0.05 for both 1-year and 5-year comparisons) (Supplementary Fig. 1). However, in the multivariable Cox regression analyses of the whole cohort, the clinical diagnosis was not associated with treatment retention for neither inefficacy nor safety discontinuations (Table 2A).

**Table 2:** Baseline factors associated with Etanercept discontinuation (multivariable Cox regression analyses)

	Discontinuation for failure	Discontinuation for adverse event
A. Baseline predictors for ETN discontinuation in all patients irrespective of diagnosis (final models)		
Diagnosis: RA (ref.)		
AxSpA	1.24 (0.84–1.83), p = 0.278	1.08 (0.59–1.95), p = 0.810
PerSpA	1.21 (0.77–1.90), p = 0.418	1.42 (0.71–2.82), p = 0.318
Gender (female versus male)	**1.99 (1.39–2.85), p < 0.001**	
Obesity (BMI > 30) (yes vs. no)	**1.46 (1.11–1.90), p = 0.006**	
RDCI	**1.18 (1.09–1.29), p < 0.001**	
Monotherapy (yes vs no)	**0.57 (0.39–0.85), p = 0.005**	
Age (per 10 years)		**1.26 (1.05–1.51), p = 0.013**
Methotrexate co-therapy (yes vs no)		**0.64 (0.42–0.99), p = 0.046**
B. Baseline predictors for ETN discontinuation due to inefficacy stratified for specific diagnosis (final models)		
	RA	SpA
Gender (female versus male)	**1.84 (1.12–3.02), p = 0.016**	**2.00 (1.15–3.45), p = 0.013**
Obesity (BMI > 30) (yes vs. no)	**1.38 (1.00–1.92), p = 0.050**	**2.93 (1.76–4.88), p < 0.001**
RDCI	**1.18 (1.06–1.31), p = 0.003**	
Swollen JC (0–28)	**1.03 (1.00–1.06), p = 0.022**	
Monotherapy (yes vs. no)		**0.53 (0.30–0.96), p = 0.036**

Numbers are Hazard Rates (95% confidence intervals). Variables also tested in regression analyses but excluded stepwise from the final models (at p > 0.10) were: symptom duration, year of etanercept start, treatment line (1st vs 2nd vs ≥ 3rd), residence (rural vs. other), number of previous csDMARDs current prednisolone use (yes/no), baseline erythrocyte sedimentation rate, physician’s global assessment and visual analogue score for pain and global. Additionally, seropositivity (RF and/or anti-CCP positive), swollen and tender joint count, and disease activity score using 28 joints (DAS28)-ESR were tested when assessing for predictors of drug retention in RA patients and baseline C-reactive protein when assessing for predictors in SpA patients. *ETN* etanercept; *RA* rheumatoid arthritis; *AxSpA* axial spondyloarthritis; *PerSpA* peripheral spondyloarthritis; *BMI* body mass index; *RDCI* rheumatic disease comorbidity index; *JC* joint count

Since inefficacy was the major cause of discontinuations, we performed adjusted analysis for factors predicting etanercept discontinuation due to treatment failure. Both in RA and SpA, treatment cessation was significantly associated with female gender and obesity (BMI > 30). Additionally, higher RDCI, and higher baseline swollen joint count predicted inefficacy discontinuations in RA, while co-administration of csDMARD(s), presumably due to co-existence of peripheral arthritis, predicted treatment failure in SpA patients (Table 2B). Significant predictors for safety-related discontinuations in the whole group were older age and no MTX co-therapy (Table 2A).

### RA patients’ long-term disease activity trajectories

**Fig. 1:**
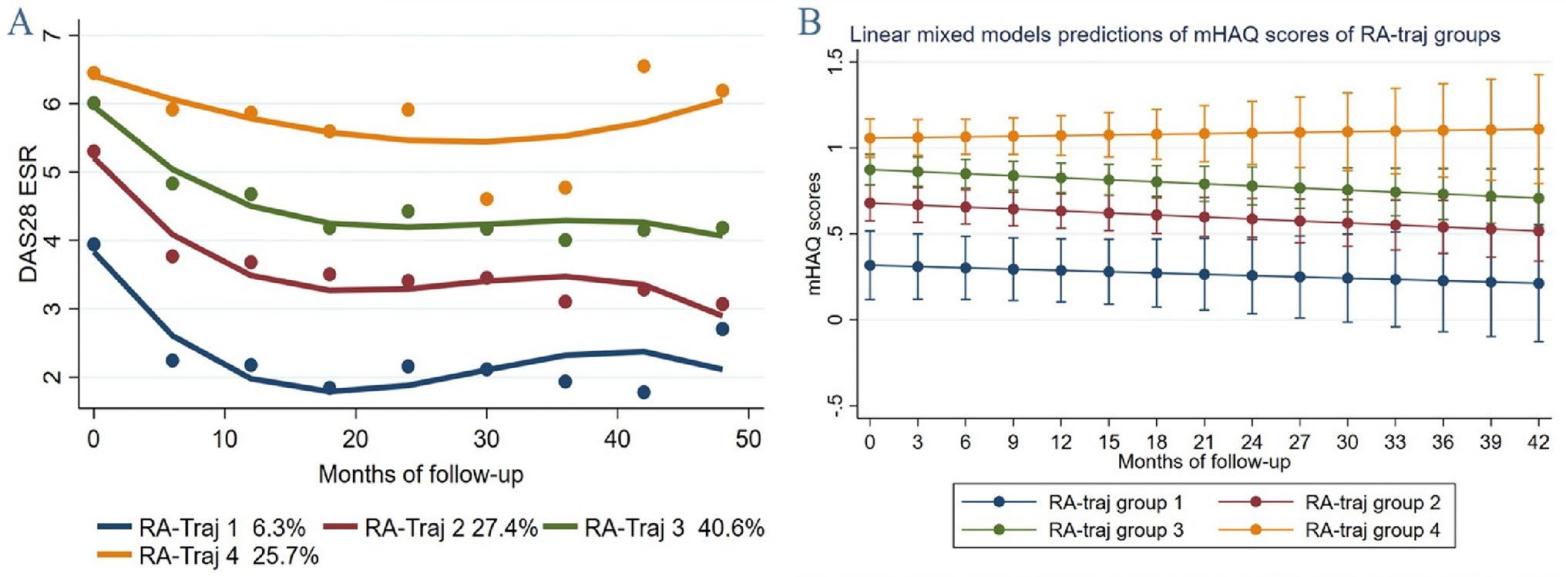
**A** Trajectory plot of disease activity levels of RA patients receiving etanercept. **B** Linear mixed models plot of mHAQ scores of RATraj groups

We furthermore aimed to categorize RA patients into discrete groups based on the long-term (4-year) course of disease activity assessed by DAS28. LCGM analysis identified 4 distinct groups of patients with similar disease activity trajectories (RATraj 1–4), clearly differentiated as early as from the 6^th^ month of etanercept initiation (Fig. 1). Patients in RATraj 1 (n = 28, 6.3% of total) improved from baseline moderate disease activity (MDA) to inactive disease, while patients in RATraj 2 (n = 123, 27.4%) and 3 (n = 182, 40.6%) improved from baseline high disease activity (HDA) to low disease activity (LDA) and MDA respectively. Improvements were gradual and plateaued after the first year of treatment. In contrast, patients in RATraj 4 (n = 116, 25.7%) remained having HDA throughout the observational period.

Univariate analysis of baseline characteristics between the four RATraj groups indicated several important differences (Table 3). In RATraj 1–2, males were overrepresented, patients had shorter disease duration, better functional status and lower total comorbidity count and RCDI index. On the contrary, RATraj 3–4 were more often obese, with higher baseline disease activity and worse physicians’ and patients’ reported scores.

**Table 3:** Between-groups univariate comparisons of baseline characteristics of RA patients classified to trajectory groups (RA-Traj 1–4) according to LCGM analysis (n = 449 included in the analysis)

	RA-Traj 1 N = 28	RA-Traj 2 N = 123	RA-Traj 3 N = 182	RA-Traj 4 N = 116	p-value
Women, N (%)	21 (75.0%)^d^	88 (71.5%)^d^	152 (83.5%)	108 (93.1%)^a,b^	** < 0.001**
Age, mean (SD)	51.2 (3.3)^b,c,d^	61.3 (1.2)^a^	60.7 (0.9)^a^	61.9 (1.2)^a^	**0.039**
Disease duration	1.2 (0.4–2.6)	2.2 (1.4–3.0)	3.9 (2.5–4.8)	2.2 (1.4–3.7)	**0.010**
RF and/or ACPA( +), N (%)	12 (42.9%)	62 (50.4%)	91 (50.0%)	62 (53.0%)	0.783
mHAQ baseline	0.25 (0.10–0.63)^b,c,d^	0.75 (0.51–1.00)^a,d^	1 (0.80–1.00)^a^	1.10 (1.00–1.20)^a,b^	** < 0.001**
Ever smoker, N (%)	11 (42.3%)	46 (47.4%)	45 (36.3%)	22 (29.7%)	0.116
Obese (BMI > 30), N (%)	5 (18.5%)^d^	41 (38.0%)	68 (45.0%)	43 (48.9%)^a^	**0.028**
Total CC	1 (0–3)^b,c,d^	3 (3–4)^a,d^	4 (3–4)^a^	4 (4–5)^a,b^	** < 0.001**
RCDI	1 (0–1)^c,d^	1 (1–1.13)^d^	1 (1–2)^a^	2 (1.98–2)^a,b^	** < 0.001**
Education > 6 years, N (%)	13 (72%)^c,d^	34 (43.5%)	37 (36%)^a^	22 (33%)^a^	*0.078*
Rural residence, N (%)	11 (44.0%)	50 (47.2%)	78 (50.1%)	54 (52.4%)	0.595
Married, N (%)	17 (73.9%)	84 (84.0%)	117 (83.6%)	74 (81.3%)	0.676
Nr of previous csDMARDs	2 (1–2)	2 (2–2)	2 (2–2)	2 (2–2.04)	0.229
Nr of previous bDMARDs	0 (0–0)	0 (0–0)	0 (0–1)	0 (0–1)	*0.085*
Treatment Nr ≤ 2, N (%)	21 (75.0%)	81 (65.9%)	100 (55.0%)	61 (52.6%)	**0.037**
MTX co-therapy, N (%)	22 (78.6%)	78 (63.4%)	110 (60.4%)	73 (62.9%)	0.329
Monotherapy, N (%)	2 (7.1%)	21 (17.1%)	25 (13.7%)	12 (10.3%)	0.343
Prednisolone yes, N(%)	8 (28.6%)	35 (28.5%)	60 (33.0%)	49 (42.2%)	0.129
DAS28 ESR	3.46 (3.13–4.14)^b,c,d^	4.92 (4.76–5.16)^a,c,d^	5.97 (5.79–6.08)^a,b,d^	6.71 (6.54–7.00)^a,b,c^	** < 0.001**
VAS global	50 (50–70)^c,d^	70 (60–70)^c,d^	80 (70–80)^a,b^	80 (80–80)^a,b^	** < 0.001**
VAS pain	50 (30–80)^c,d^	70 (60–70)^c,d^	80 (70–80)^a,b^	80 (80–80)^a,b^	** < 0.001**
VAS physician	63 (50–73)^b,c,d^	75 (74–75)^a,d^	75 (75–75)^a,d^	75 (75–75)^a,b,c^	** < 0.001**
CRP	0.30 (0.30–0.31)^b,c,d^	0.41 (0.30–0.54)^a^	0.40 (0.32–0.60)^a^	0.48 (0.32–0.80)^a^	**0.023**
ESR	11 (6.0–15.0)^b,c,d^	21 (19.7–25-7)^a,d^	25 (22.8–29.2)	35 (32.5–40.0)^a,b,c^	** < 0.001**
IR for SAEs	1.91 (0.27–13.6)	1.75 (0.72–4.20)	4.81 (2.95- 7.86)	11.5 (7.13- 18.46)	** < 0.001**

Variables are reported as Median (95% Confidence Interval for the Median) unless otherwise specified. *IR* Incidence rate; *SAEs* serious adverse events. For other definitions see Table 1

^a^A significant difference from RA-Traj group 1

^b^A significant difference from RA-Traj group 2

^c^A significant difference from RA-Traj group 3

^d^A significant difference from RA-Traj group 4

Disease activity significantly contributes to patient’s functional status. Thus, we assessed whether the four trajectory groups presented above, correspond to distinct long-term courses of function (based on mHAQ values). Indeed, results from the linear mixed models indicated statistically significant differences in mHAQ scores between the four RATraj groups since baseline which were preserved over time. RATraj groups 1–3 presented a small yet steady improvement in mHAQ scores, contrary to RATraj group 4 in which functional status worsened over time (Fig. 1).

Furthermore, we investigated the impact of the different disease activity trajectory groups on the incidence of serious adverse events. Interestingly, patients in RATraj 3 and 4 experienced significantly more serious adverse events (SAEs) during follow-up compared to RATraj 1 and 2 (Incidence Rate (IR) for SAEs 11.5/100 patient-years vs 1.91/100 patient-years in RATraj 4 and RATraj 1 respectively, p < 0.001) (Table 3).

### Distinct long-term disease activity trajectories for AxSpA patients

**Fig. 2:**
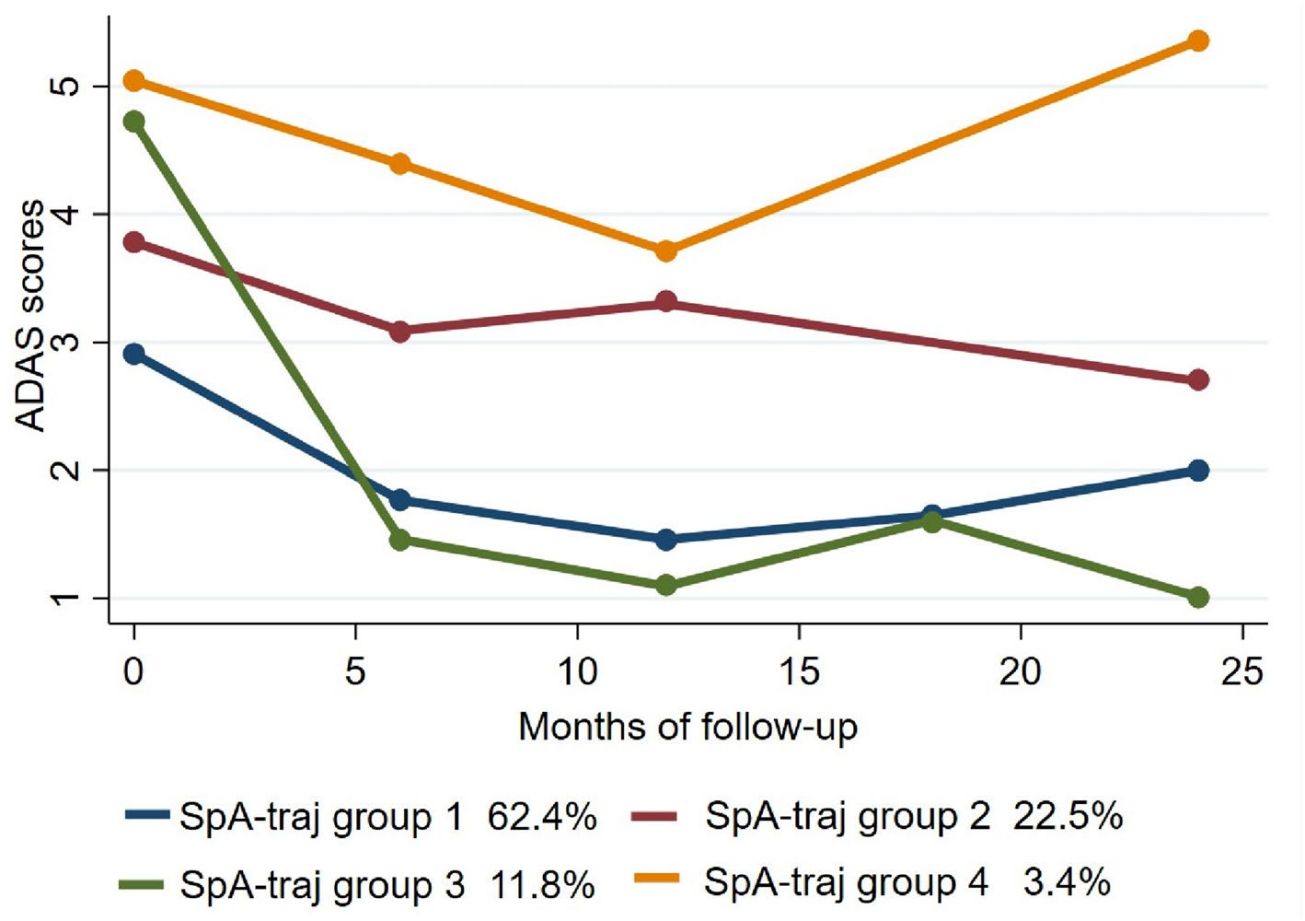
Trajectory plot of disease activity levels of AxSpA patients receiving etanercept

We also assessed for 2-year trajectories of AxSpA patients based on disease activity quantified by the ASDAS-CRP. Applying LCGM, we found that patients were grouped in four distinct trajectories (SpATraj 1–4), again as early as from 6^th^ month of etanercept treatment (Fig. 2). Approximately 74% of the patients were grouped in SpATraj 1 (n = 111, 62.4%) and 3 (n = 21, 11.8%) experiencing fast improvement in their disease activity from HDA or very HDA to LDA and inactive disease respectively and steadily remained at these levels for the total follow-up period. One out of four patients were grouped in either SpATraj 2 (n = 40; 22.5%), in which there was a small clinical improvement, but residual high disease activity remained, or in SpATraj 4 (n = 6, 3.4%), in which patients were absolutely non-responders.

Several baseline patient parameters were different across the four AxSpA trajectories groups (Table 4). Univariate analysis between SpATraj groups’ baseline characteristics indicated that patients in SpATraj groups 1&3 (responders) were more frequently males, less often obese, with fewer comorbidities and lower RCDI. Patients in SpATraj group 1 had shorter disease duration, more often received etanercept as monotherapy and fewer of them received prednisolone. Furthermore, participants classified in SpATraj group 1 had lower baseline disease activity levels (ASDAS, VAS global, VAS pain) and lower levels of acute phase reactants (ESR, CRP). SpATraj group 3 patients, who also responded to treatment, had significantly higher CRP values than patients in other groups.

**Table 4:** Between-groups univariate comparisons of baseline characteristics of AxSpA patients classified to trajectory groups (SpA-Traj 1–4) according to LCGM analysis (n = 178)

	SpA-Traj 1 N = 111	SpA-Traj 2 N = 40	SpA-Traj 3 N = 21	SpA-Traj 4 N = 6	p-value
Women, N (%)	32 (28.8%)^b^	23 (57.5%)^a,c,d^	5 (23.8%)^b^	2 (33.3%)^b^	**0.008**
Age, mean (SD)	45.3 (12.1)	50.2 (13.3)	44.3 (13.1)	51.4 (16.4)	0.119
Disease duration	0.62 (0.17–1.20)^b^	2.03 (0.88–9.06)^a^	3.15 (0.04–10.5)	5.85 (1.37–21.8)	**0.006**
Ever Smoker, N (%)	50 (69.4%)	20 (80.0%)	11 (73.3%)	3 (60.0%)	0.708
Obese (BMI > 30), N (%)	12 (15.3%)	11 (40.7%)	3 (17.7%)	1 (33.3%)	**0.046**
Total CC	1 (1–2)^b^	3 (2–5)^a^	2 (1–3)	3 (1–4)	**0.003**
RCDI	0 (0–1)	1 (1–2)^c^	0 (0–1)^b^	0 (0–1)	**0.043**
Nr of previous csDMARDs	1 (0–1)	1 (1–1.7)	1 (0–2)	1 (0–2)	0.079
Nr of previous bDMARDs	0 (0–1)	1 (0–1)	1 (0.5–1)	1 (0–2)	0.383
Treatment Nr ≤ 2, N (%)	56 (50.5%)	19 (47.5%)	6 (28.6%)	2 (33.3%)	0.279
MTX co-therapy, N (%)	32 (28.8%)^b,c^	22 (55.0%)^a,d^	10 (47.6%)^a,d^	1 (16.7%)^b,c^	**0.013**
Monotherapy, N (%)	75 (67.5%)^b^	13 (32.5%)^a,d^	9 (42.6%)^a,d^	4 (66.7%)^b,c^	**0.001**
Prednisolone yes, N(%)	8 (8.1%)^d^	7 (17.5%)^d^	3 (14.3%)^d^	3 (50.0%)^a,b,c^	**0.013**
ASDAS-CRP	2.94 (2.70–3.15)^b,c^	3.77 (3.65–4.05)^a,c^	4.70 (4.52–5.17)^a,b^	5.09 (4.95–5.46)	** < 0.001**
VAS global	60 (50–70)^b,c^	80 (80–85)^a^	85 (80–90)^a^	80 (70–100)	** < 0.001**
VAS pain	60 (50–70)^b,c^	80 (70–80)^a,c^	90 (80–95)^a,b^	80 (60–100)	** < 0.001**
VAS physician	75 (75–80)^c^	75 (75–80)	75 (75–88)^a^	75 (55–75)	0.105
CRP	0.77 (0.50–1.57)^c^	0.94 (0.40–1.35)^c^	3.95 (1.34–7.75)^a,b^	1.12 (0.30–8.00)	**0.007**
ESR	18 (15–20)^c,d^	30 (19–37)^c^	58 (50–84)^a,b^	69 (15–90)^a^	**0.001**
IR for SAEs	1.57 (0.39–6.31)	5.50 (3.31- 9.12)	1.77 (0.24–12.59)	3.94 (0.98–15.79)	** < 0.001**

Variables are reported as Median (95% Confidence Interval for the Median) unless otherwise specified. IR: Incidence rate; SAEs: Serious adverse events. For other definitions see Table 1

^a^A significant difference from SpA-Traj group 1

^b^A significant difference from SpA-Traj group 2

^c^A significant difference from SpA-Traj group 3

^d^A significant difference from SpA-Traj group 4

Notably, patients responding to etanercept treatment (SpATraj 1 and 3) experienced also less SAEs during follow-up than patients in SpA Traj 2 and 4 (non-responders) (IR for SAEs 5.5/100 patient-years vs 1.6/100 patient-years in SpATraj 2 and SpATraj 1 respectively, p < 0.001; Table 4).

## Discussion

RA and AxSpA are heterogenous diseases concerning pathophysiology, phenotypes and response to treatment-prognosis [32–35]. Most studies have characterized treatment responses of biologic therapies at the group level, unifying all patients and reporting on average disease activity evolution. In the present study we identified 4 long-term disease activity trajectories in both RA and AxSpA patients initiating etanercept therapy in real-world clinical practice and we investigated the baseline characteristics of the patients who follow different disease activity courses. We also compared etanercept retention in patients with either RA and spondyloarthritis and we identified predictors for long-term etanercept survival in the whole cohort and separately for RA and AxSpA.

The major strength of the present study is the large cohort of unselected patients starting etanercept. Patients were prospectively monitored using the same protocol in a tertiary rheumatology center. Disease activity trajectories, as well as treatment response and survival may differ by treatment class and stage of disease. As all patients in this cohort had established disease and received the same main treatment, we consider that trajectories found in the present analysis could represent “true” disease-related courses in a cohort of patients treated with etanercept.

In RA patients, we were able to define four distinct latent disease activity-related subgroups during the course of treatment. Patients in traj. groups 1&2 (33.7% of total) showed a significant clinical improvement from the first 6 months of therapy and remained in remission or low disease activity levels for up-to 48 months of treatment. In 40.6% of long-term etanercept treated patients (RA-Traj group 3) there was only a partial response to therapy and patients had moderate disease activity throughout observation time, while patients in RA-Traj group 4 (25.7% of total) had only limited, if any, response. In this latter group, women were overrepresented, patients were older, had experienced more bDMARDs failures and had more comorbidities. Patients on RA-Traj groups 1 & 2 had shorter disease duration, lower comorbidities count and started etanercept treatment with lower disease activity. These factors are similar to predictors of treatment response in other long-term prospective studies and national registries [36, 37].

Most of previous studies have also shown distinct disease activity trajectories in early as well as in established RA under therapy with different biologics [10, 11, 38]. Differences in described trajectories between the aforementioned studies can be explained by methodological differences, heterogenous populations (early/established RA) and different background therapies. Our data together with the previous reports characterizing heterogenous subgroups of inflammatory burden of RA [10, 11], further corroborate “molecular” heterogenicity of the disease depicted by RNAseq profiling of synovium and peripheral blood, which predicted clinical responses and radiological outcome [33, 39].

It has been previously shown that RA patients on persistent moderate disease activity had more serious adverse events as compared to patients in lower disease activity levels [40], a finding confirmed in the present analysis. Indeed, patients in RA-Traj groups 1&2 with a better long-term control of disease activity experienced less SAEs as compared to RATraj 3 & 4. We consider that patients with persistently higher inflammatory burden are predisposed to a higher frequency of serious infections (a major contributor to SAEs) as well as cardiovascular SAEs, or medication side effects. An additional explanation could be that responders are generally a more “healthy” group according to their baseline characteristics: they are younger, with better functional status, less frequently obese, with lower total number of comorbidities and receive less frequently corticosteroids. On the other hand, function, as measured with HAQ, improved in all groups except in RA-Traj group 4 patients, who remained having HDA and had worsening function course over time (Fig. 1).

Regarding AxSpA, disease activity “endotypes” have been even less characterized. Herein, we have identified 4 distinct disease activity trajectories, based on ASDAS-CRP evolution over 2 years (Fig. 2). We consider that subgroups identified represent valid AxSpA disease activity grouping, since 74% of the patients (AxSpA-Traj groups 1&3) showed significant clinical responses maintained for the 2 years of follow-up, a percentage rather comparable to TNFis retention rate reported by others [41]. Subgroups defined by our analysis in an established AxSpA cohort treated by the same TNFi, seem to be at least partially comparable to those described in a mixed AxSpA population of early and established cohorts on different background therapies [15]. Unfortunately, data assessing functional status in our AxSpA cohort were limited, and thus we could not correlate disease activity trajectories to long-term functional outcomes. Our data further support that females, obesity, disease duration, baseline disease activity and comorbidities’ accumulation are associated with an adverse inflammatory burden course (Table 4). Interestingly most of these parameters were also associated to an adverse outcome in the study of Imkamp et al. [15] and in large registry-based studies [42]. Female gender has been associated with higher incidence of fibromyalgia and higher PROs, thus resulting to less response in several studies [43]. Similarly, disease duration may be associated with accrual of damage and thus worse outcomes while accumulation of comorbidities may affect perception of pain, limit drug options and restrict function. Nevertheless, causality studies or mechanistic studies assessing how the aforementioned factors affect clinical responses are lacking.

Latent class trajectory modeling has been successfully applied before, revealing the existence of several distinct trajectory groups [11]. Literature has pointed out the possible existence of 3–7 distinct groups according to the methodology and outcome variables used in each case. We repeated our trajectory analysis for various number of groups (ranging from two to seven groups) and we chose the best fitting model (which consisted of four trajectory groups) based on the lowest value of BIC. Although results from trajectory analyses should always be interpreted with caution [44], our findings seem to be in accordance with the results produced from drug-survival analysis in terms of the overall drug performance.

Finally, we compared long-term etanercept survival between the three diagnoses, RA, AxSpA and PerSpA. Although in crude analysis patients with RA showed a lower drug survival, in the multivariate analysis after adjustments for several different baseline factors, diagnosis was not a predictor of etanercept discontinuation. This finding, which has been also shown by other groups for golimumab and certolizumab [45, 46], may be considered contradictory with the common perception that survival of TNFis is higher in SpA than in RA patients; nevertheless, it further supports the validity of proper statistical analysis and comparisons between groups in the same study as compared to crude disease-based analysis. Approximately 57.5% (22%), 67% (30%) and 56.8% (21%) of RA, AxSpA and PerSpA patients respectively remained on etanercept at 1 year (5 years). These drug survival rates are lower than what is reported by other registries [47]. However, considering that etanercept was the ≥ 2nd bDMARD in approximately half of the patients in our study, these data are comparable to those reported by other groups both for RA and for SpA [48, 49].

Owing to the observational design of the study, some co-variates were missing and some patients were lost to follow-up. To address this limitation we tried to triangulate our results using both survival analysis, which is a robust method for right-censored data with “non-informative” censorship [50], and latent class growth models. We further included only baseline variables with < 30% missing values in the univariate and multivariate Cox-regression models, as use of completer-analysis may have lowered the generalizability of our results. Furthermore, despite the relatively large overall size of our cohort, the number of patients with SpA—especially peripheral SpA—was low therefore our findings should be interpreted with caution. Moreover, AxSpA patients with available data for the longitudinal analysis were rather limited, and trajectories analysis in this group was performed only for the first two years of follow-up.

DAS28 index was used in our analyses, although SDAI and CDAI may better reflect disease activity in RA. However, CRP and physician’s global assessment needed for these indices is missing in several cases in our dataset, resulting in a lower number of available patients for analysis. A sensitivity analysis performed to model disease activity trajectories based on CDAI identified 3 trajectory groups, while the baseline variables differentiating the groups at baseline were comparable to those shown in the analysis based on DAS28 (data not shown).

Concluding, the present study is the first trajectory modelling analysis of etanercept-treated patients with RA/AxSpA in the same prospective research setting. We identified patients with different responsesand prognosis over time in respect to function and serious adverse events. Male gender, lower disease activity and fewer comorbidities, were found to predict generally a more favorable outcome. These factors could assist rheumatologists for a more personalized treatment approach.

### Supplementary Information

Below is the link to the electronic supplementary material.

Supplementary file1 (PDF 76 KB)

Supplementary file2 (PDF 454 KB)

## Data Availability

The data and analytic methods that support the findings of this study are available to qualified investigators upon request to the corresponding author.
