# Supplementary material for: Distinct long-term disease activity trajectories differentiate early on treatment with etanercept in both rheumatoid arthritis and spondylarthritis patients: a prospective cohort study
Source: Rheumatol Int. 2023 Oct 10;44(2):249–61. doi: 10.1007/s00296-023-05455-7 (PMC10796740; doi:10.1007/s00296-023-05455-7)
Supplement: Supplementary file 1 — Supplementary file1 (PDF 76 KB) [file 296_2023_5455_MOESM1_ESM.pdf]

SUPPLEMENTARY TABLES

**Supplementary Table 1.** Etanercept discontinuations and reasons thereof, during whole follow-up time, N (% of patients who started Etanercept)				
	**Total**	**RA**	**AxSpA**	**PerSpA**
**Total**	466 (65.5)	311 (69)	96 (54)	59 (71)
**Inefficacy**	327 (46)	232 (52)	60 (34)	35 (42)
**Adverse events**	87 (12)	56 (12)	20 (11)	11 (13)
**Other**	52 (7)	23 (5)	16 (9)	13 (16)

**Supplementary Table 2.** Type and number of adverse events in Etanercept patients' cohort [N (events/100 patient-years of follow-up)]				
	**Diagnosis**			
	**Total**	**RA**	**AxSpA**	**PerSpA**
**All**	**490 (36)**	**277 (34)**	**137 (37)**	**76 (42)**
**Serious**	**90 (6.6)**	**52 (6.3)**	**26 (7.0)**	**12 (6.6)**
**Infections**	**36 (2.6)**	**21 (2.6)**	**10 (2.7)**	**5 (2.8)**
Respiratory	12 (0.9)	9 (1.1)	2 (0.5)	1 (0.6)
Gastrointestinal	8 (0.6)	3 (0.4)	4 (1.1)	1 (0.6)
Genitourinary	6 (0.4)	3 (0.4)	2 (0.5)	1 (0.6)
SSTI	4 (0.3)	2 (0.2)	0 (0)	2 (1.1)
Musculoskeletal	3 (0.2)	2 (0.2)	1 (0.3)	2 (1.1)
**Cardiovascular events**	**14 (1.0)**	**11 (1.3)**	**3 (0.8)**	**0 (0)**
**Tumors**	**9 (0.7)**	**5 (0.6)**	**3 (0.8)**	**1 (0.6)**
**Gastrointestinal/Hepatobiliary**	**6 (0.4)**	**1 (0.1)**	**5 (1.4)**	**0 (0)**
**Musculoskeletal**	**6 (0.4)**	**3 (0.4)**	**0 (0)**	**3 (1.2)**
**Nervous**	**5 (0.4)**	**2 (0.2)**	**2 (0.5)**	**1 (0.6)**
**Moderate**	**216 (16)**	**131 (16)**	**57 (15)**	**28 (15.5)**
Infections	126 (9.2)	78 (9.5)	29 (7.8)	19 (10.5)
Respiratory	81 (5.9)	51 (6.2)	15 (4.0)	15 (8.3)
SSTI	20 (1.6)	10 (1.2)	7 (1.9)	3 (1.2)
Genitourinary	14 (1.0)	8 (1.0)	5 (1.4)	1 (0.6)
Injection reactions	16 (1.2)	9 (1.1)	6 (1.6)	1 (0.6)
**Mild**	**183 (13)**	**94 (11.5)**	**54 (14.6)**	**35 (19.3)**
Infections	87 (6.3)	40 (4.9)	22 (5.9)	25 (13.8)
Injection reactions	39 (2.8)	24 (2.9)	11 (3.0)	4 (2.2)
Numbers are absolute numbers of events (events/100 patient-years of follow-up)				
