# Supplementary material for: Distinct long-term disease activity trajectories differentiate early on treatment with etanercept in both rheumatoid arthritis and spondylarthritis patients: a prospective cohort study
Source: Rheumatol Int. 2023 Oct 10;44(2):249–61. doi: 10.1007/s00296-023-05455-7 (PMC10796740; doi:10.1007/s00296-023-05455-7)
Supplement: Supplementary file 2 — Supplementary file2 (PDF 454 KB) [file 296_2023_5455_MOESM2_ESM.pdf]

Supplementary Figure 1. Treatment retention (A) for all reasons of discontinuation (inefficacy, adverse events and other), and (B) only for inefficacy discontinuations, stratified by diagnosis**.**

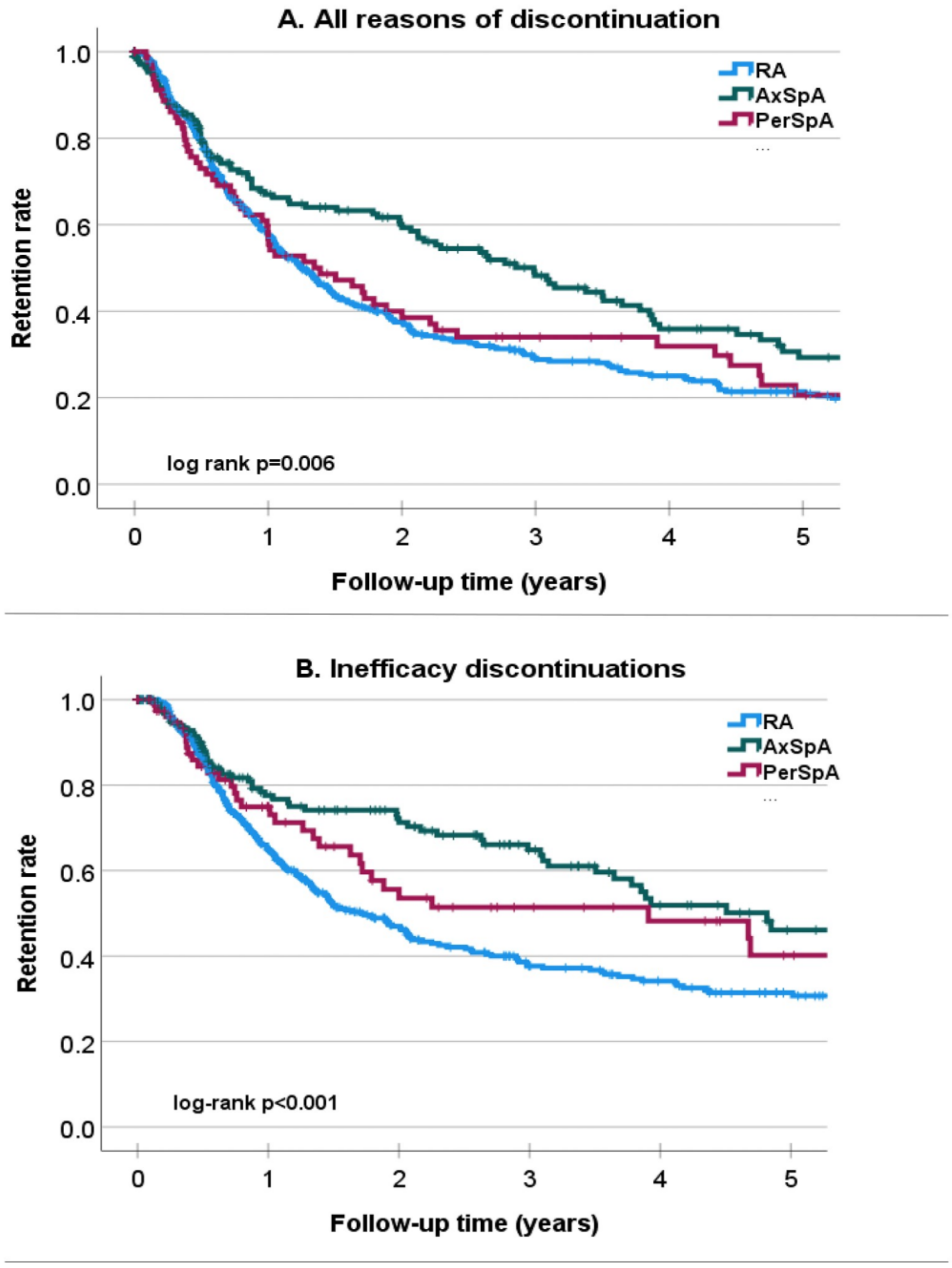

Numbers at risk [all discontinuations]

RA	450 [176]	221 [71]	120 [25]	80 [11]	64 [9]	43
AxSpA	178 [51]	92 [9]	75 [14]	52 [12]	32 [5]	21
PerSpA	83 [33]	42 [12]	27 [4]	19 [1]	15 [2]	9
